# Supplementary material for: National Disparities in Antibiotic Prescribing by Race, Ethnicity, Age Group, and Sex in United States Ambulatory Care Visits, 2009 to 2016
Source: Antibiotics (Basel). 2022 Dec 28;12(1):51. doi: 10.3390/antibiotics12010051 (PMC9854843; doi:10.3390/antibiotics12010051)
Supplement: Supplementary file 1 [file antibiotics-12-00051-s001.zip › antibiotics-2106920-supplementary.pdf]

Supplementary Materials

Table S1. Definitions for antibiotics and antibiotic classes

Antibiotic Class	Antibiotics	Multum Code(s)
Fluoroquinolones	Ciprofloxacin	d00011
	Levofloxacin	d04109
	Moxifloxacin	d04500
Beta-lactams	Penicillin VK	d07730
	Amoxicillin	d00088
	Dicloxacillin	d00153
BL/BLI	Amoxicillin/clavulanate	d00089
Cephalosporins	Cefadroxil	d00080
	Cephalexin	d00096
	Cefaclor	d00081
	Cefprozil	d00073
	Cefuroxime	d00056
	Cefdinir	d04256
	Cefixime	d00072
	Cefpodoxime	d00095
	Ceftibuten	d03874
Macrolides	Azithromycin	d00091
	Clarithromycin	d00097
	Erythromycin	d00046
Rifamycins	Rifampin	d00047
	Rifabutin	d01097
	Rifapentine	d04327
Tetracyclines	Doxycycline	d00037
	Minocycline	d00110
	Tetracycline	d00041
Other	Clindamycin	d00043
	Metronidazole	d00108
	Nitrofurantoin	d00112
	Sulfamethoxazole-Trimethoprim	d00124, d00119

BL/BLI: beta lactam/beta-lactamase inhibitor

Table S2. Diagnosis definitions

Diagnosis	ICD-9-CM Code(s)	ICD-10 Code(s)
**Antibiotics almost always indicated**		
Pneumonia	481-486	J13-J18
Urinary tract infection	590.1, 590.2, 590.8, 590.9, 595.0, 595.9, 599.0	N10, N12, N15, N30, N39
Other bacterial infections	010-018, 020-027, 030-033, 036-041, 070-104, 130-139, 320-323, 383, 475	A10-A49, A54-58, A63, 64, A70-79<br>B95-96, B99, H70, H95, J36
**Antibiotics may be indicated**		
Acne	706.0, 706.1	L70
Gastrointestinal infections	001-009, 787, 789	A00-A05, A09, R11, R15, K67
Pharyngitis	462, 463, 034	J02, J03
Sinusitis	461, 473	J01, J32
Skin infections	680-686, 035, 110-111, 704.8, 728.0, 611.0, 771.5, 728.86, 380.0-380.1	L00-08, L66, L73, L88, L89, L98<br>K12, E83, M60, N61, P39, M72, H60-H62
Suppurative otitis media	382	H66
**Antibiotics not indicated**		
Asthma/allergy	493, 477, 995.3	J45, J30, T78.4
Bronchitis	490, 466	J40, J20-21
Influenza	487, 488	J09-J11
Non-suppurative otitis media	381	H65, H67
Viral pneumonia	480	J12
Viral upper respiratory tract infections	460, 464, 465, 786.2	J00, J04, J06, R05
Other gastrointestinal conditions	520-579	K00-14, K20-31, K35-38, K40-46, K50-52, K55-64, K65, K66, K68, K70-77, K80-87, K90-95
Other skin conditions	690-698, 700-709, 870-897, 910-919, 940-949, 360-379, 380-389 (excluding 380.0-380.1, 381, 382, 383), 782, 785.4, 785.6	L10-14, L20-30, L40-45, L49-54, L60-65, L67-72, L74-75, L80-87, H68, 69, H71-H75, R17, R20-23, I96, R59
Other genitourinary conditions	580-629 (excluding UTI codes and 611.0), 788.1	N00-N08, N16, N17-N19, N20-23, N25-29, N30-38, N40-53, N62-N65, N70-N77, N80-N98, N99, R30
Other infections	042, 045-049, 050-059, 060-069, 112-129	A06-A08, A50-53, A59-60, A65-69, A80-B94, B97

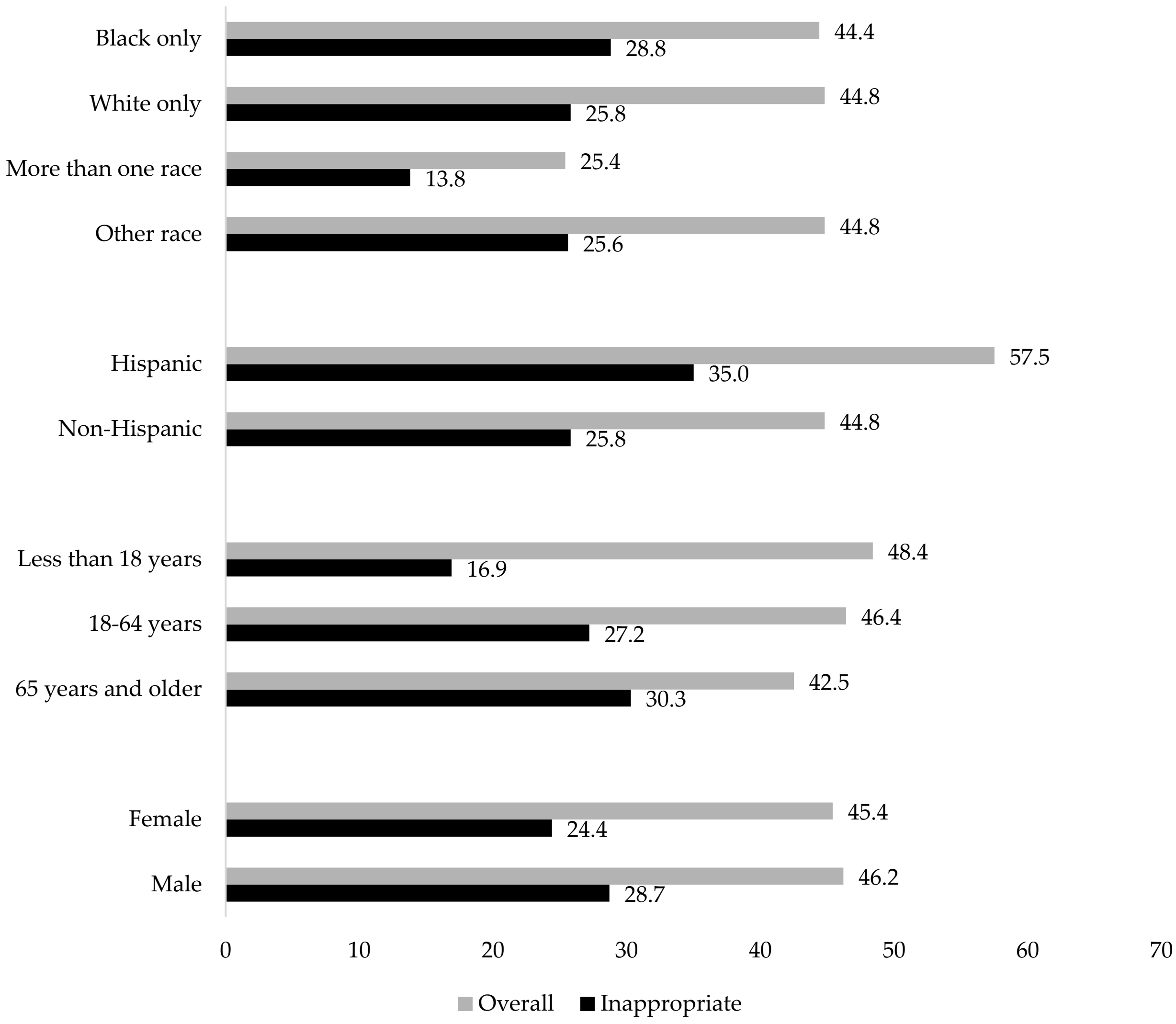

Figure S1. Rate per 1,000 total visits of patients prescribed broad-spectrum antibiotics by patient subgroup. **Note:** $p<0.0001$ for all comparisons of overall and inappropriate antibiotic prescribing rates for each subgroup

Table S3. Most common diagnoses where inappropriate antibiotics were prescribed by subgroup

	Other skin conditions	Viral upper respiratory tract infection	Bronchitis
**Overall, %**	17.3	13.3	11.2
**Race, %**			
Black only	14.7	11.6	7.4
White only	17.3	13.6	11.6
More than one race	17.2	11.7	6.7
Other	20.2	12.2	17.9
**Ethnicity, %**			
Hispanic	14.5	13.9	7.9
Non-Hispanic	16.9	13.2	11.6
**Age group, %**			
Less than 18 years	20.5	26.4	15.2
18 to 64 years	15.4	11.5	11.3
65 years and older	19.1	8.6	8.4
**Sex, %**			
Female	16.6	13.4	10.5
Male	18.2	13.2	12.2
